# The association of pre-operative biomarkers of endothelial dysfunction with the risk of post-operative neurocognitive disorders: results from the BioCog study

**DOI:** 10.1186/s12871-024-02722-3

**Published:** 2024-10-08

**Authors:** Sara Moazzen, Jürgen Janke, Arjen J. C. Slooter, Georg Winterer, Claudia Spies, Tobias Pischon, Insa Feinkohl

**Affiliations:** 1 Max-Delbrueck-Center for Molecular Medicine in the Helmholtz Association (MDC), Molecular Epidemiology Research Group, Berlin, Germany; 2 Max-Delbrueck-Center for Molecular Medicine in the Helmholtz Association (MDC), Biobank Technology Platform, Berlin, Germany; 3 Departments of Psychiatry and Intensive Care Medicine, UMC Utrecht Brain Center, University Medical Center Utrecht, Utrecht University, Utrecht, the Netherlands; 4 Department of Neurology, UZ Brussel and Vrije Universiteit Brussel, Brussels, Belgium; 5 Charité - Universitätsmedizin Berlin, corporate member of Freie Universität Berlin and Humboldt-Universität Zu Berlin, Berlin, Germany; 6 PI Health Solutions GmbH, Berlin, Germany; 7 Medical Biometry and Epidemiology Research Group, Witten/Herdecke University, Witten, Germany

**Keywords:** Ageing, Surgery, Endothelial function, Postoperative delirium, Postoperative cognitive dysfunction, Cognitive dysfunction, Postoperative cognitive complications, Neuropsychological tests, Endothelial dysfunction, Biomarkers

## Abstract

**Introduction:**

Endothelial dysfunction (ED) promotes the development of atherosclerosis, and studies suggest an association with age-related neurocognitive disorders. It is currently unclear whether ED is also associated with the risk of perioperative neurocognitive disorders.

**Method:**

We included 788 participants aged ≥ 65 years of the BioCog study. Patients were scheduled to undergo elective surgery with expected duration > 60 min. Blood was collected before surgery for measurement of 5 biomarkers of ED: asymmetric and symmetric dimethylarginine (ADMA; SDMA), intercellular and vascular adhesion molecule (ICAM-1, VCAM-1), and von Willebrand factor (vWF). Patients were monitored for the occurrence of postoperative delirium (POD) daily until the 7th postoperative day. 537 (68.1%) patients returned for a 3-month follow-up. Post-operative cognitive dysfunction (POCD) was defined from the change in results on a battery of 6 neuropsychological tests between baseline and 3 months, compared to the change in results of a control group during the 3-month interval. The associations of each of the 5 ED biomarkers with POD and POCD respectively were determined using multiple logistic regression analyses with adjustment for age, sex, surgery type, pre-morbid IQ, body mass index, hypertension, diabetes, HbA1C, triglyceride, total and HDL cholesterol.

**Results:**

19.8% of 788 patients developed POD; 10.1% of 537 patients had POCD at 3 months. Concentrations of ED biomarkers were not significantly associated with a POD. A higher VCAM-1 concentration was associated with a reduced POCD risk (adjusted odds ratio 0.55; 95% CI: 0.35–0.86). No further statistically significant results were found.

**Conclusion:**

Pre-operative concentrations of ED biomarkers were not associated with POD risk. We unexpectedly found higher VCAM-1 to be associated with a reduced POCD risk. Further studies are needed to evaluate these findings.

**Supplementary Information:**

The online version contains supplementary material available at 10.1186/s12871-024-02722-3.

## Introduction

Around 313 million surgeries are performed annually worldwide [1] and, particularly in older adults, they are often accompanied by perioperative neurocognitive disorders. Post-operative delirium (POD) is a clinical diagnosis characterized by a disturbance of attention, consciousness, perception, and cognition [2, 3]. POD occurs in a substantial proportion of older patients within the first days following surgery [4]. Post-operative cognitive dysfunction (POCD) is defined as a decline in neuropsychological test performance between pre- and post-operative assessment [5]. The methods used to define POCD are extremely heterogeneous, complicating comparisons across studies [6]. Three to six months after surgery, POCD has been described in 10% to 25% of patients [7]. Both POD and POCD may be associated with premature mortality [8–10] and reduced quality of life [11, 12], making them important public health issues that warrant investigation. In the epidemiology of POD and POCD, a number of risk factors have been identified to date, including advanced age, pre-existing neurocognitive disorders, and metabolic factors [13–15].

The endothelium plays a major role in cardiovascular homeostasis, functioning both as the barrier and the bridge between blood, the vascular wall, and surrounding tissues. It controls the exchange of biomolecules, signaling chemicals, and nutrients [16]. A healthy endothelium ensures that blood is delivered to all parts of the vascular tree [17] and regulates platelet adhesion [18]. Endothelial dysfunction (ED), on the other hand, is characterized by the deposition of lipids, fatty streaks, and lipid-rich atherosclerotic plaques [18]. ED additionally leads to atherosclerosis through nitric oxide (NO) depletion, which in turn promotes inflammation at the endothelium, setting the body into a pro-inflammatory state. The latter can become exacerbated by surgery with trial evidence in fact to suggest a potential causal effect on cognitive risk [19–21]. The brain has a large endothelial surface [22]. Its function can therefore likely be compromised by prolonged systemic ED.

Given the fact that the endothelium is a complex system, a panel of markers could be reflective of endothelial physiology and pathology [23]. Known biomarkers of systemic ED include asymmetric and symmetric dimethylarginine (ADMA, SDMA), intercellular adhesion molecule 1 (ICAM-1), and vascular cell adhesion molecule 1 (VCAM-1) as well as von Willebrand factor (vWF). ADMA is known as an inhibitor of NO synthase. SDMA is a regioisomer of ADMA; however, does not inhibit the synthesis of NO. ICAM-1 and VCAM-1 promote the adherence of leukocytes and the influx of macromolecules through the vessel wall [24]. Finally, vWF is a protein involved in the coagulation pathway [25]. Elevated concentrations of the biomarkers above in circulation, which all tend to correlate positively with one another, are indicative of systemic ED.

Concentrations of circulating ED biomarkers have been linked to brain degenerative processes that manifest in age-related neurocognitive disorders [26–28] including dementia [29–31]. They may additionally correlate with the severity of brain disease [29, 32–35]. It is unclear whether ED is also related to perioperative neurocognitive disorders. To the best of our knowledge, no study has reported on ED and POCD and three studies on ED and POD risk have produced conflicting results [36–38]. These studies have also been limited by small sample sizes and have focused on individual ED biomarkers rather than concurrently assessing several ED biomarkers for direct comparison and evaluation of their interdependence in the associations with POD. On the other hand, previous findings indicate an association between elevated levels of ED biomarkers with the presence or progression of white matter hyperintensities (WMH) [39, 40] and white matter lesions [29, 41–43], while white matter lesions are associated with an increased risk of POD [44] and POCD [45]. These findings suggest a possible mediatory role for the presence of WMH and white matter lesions in the hypothesized association of ED with POD and POCD risk.

Here, we used data from a large cohort of older surgical patients firstly to investigate the association of pre-operative concentrations of ED biomarkers in circulation with the risk of i) POD during the hospital stay and ii) POCD at 3-month follow-up. We compared results for five biomarkers (ADMA, SDMA, ICAM-1, VCAM-1, and vWF) and determined the interdependence in their relationships with POD/POCD. We additionally adjusted for potential confounding and, separately, for mediating factors, including the presence of pre-operative WMH, and white matter lesions. We hypothesized that higher pre-operative concentrations of the 5 ED biomarkers are associated with an increased POD and POCD risk and that the respective association may in part be driven by WMH and white matter lesions (such that statistical adjustment for these factors would lead to statistical non-significance).

## Method

### Study design

We used data and biomaterial from the Biomarker Development for Postoperative Cognitive Impairment in the Elderly (BioCog) study [46]. BioCog is an EU-funded prospective cohort study that tracked the cognitive development of older surgical patients who had been recruited between 2014 and 2017 in Utrecht, the Netherlands, and Berlin, Germany (for full inclusion and exclusion criteria, see [47]). In brief, patients were ≥ 65 years old, Caucasian, and scheduled to undergo elective surgery of any type (e.g., orthopedic, abdominal, peripheral surgery) with expected duration ≥ 60 min under regional, local or general anesthesia. Exclusion criteria were (among others) Mini-Mental State Examination (MMSE) ≤ 23 and presence of a neuropsychiatric disease affecting the ability to perform cognitive tests. Patients underwent cognitive and clinical examination including magnetic resonance imaging (MRI) during the days before surgery. They were followed up during the hospital stay until 7 days after surgery/discharge and returned to the clinic for follow-up at 3 months. 933 participants met the inclusion criteria and were included in the BioCog cohort (see Supplementary Fig. 1).

### Pre-operative sociodemographic, clinical, and cognitive assessment

Sociodemographic information and medical history including history of diabetes, transient ischemic attack (TIA), and stroke, were self-reported. Clinical assessments included the measurement of height and weight for the calculation of body mass index (BMI) and the measurement of systolic and diastolic blood pressure. Pre-morbid intelligence quotient (IQ) was measured using the German version of the Mill-Hill vocabulary test (German patients) and the Dutch adult reading test (Dutch patients). Scores on both scales were converted to IQ based on published norms and merged to derive a single variable ‘pre-morbid IQ’. Pre-operative cognitive assessments of 'fluid ability' (further outlined below) were used to calculate a *g *factor of global cognitive ability for each patient. 

### Pre-operative blood collection and routine lab analysis

Blood was collected following an overnight fast immediately before surgery and shipped to immediate labs adjacent to the respective hospital site. Samples were additionally stored at -80° in a central biobank. Routine laboratory parameters were measured in serum (triglycerides, high-density lipoprotein, HDL-C, total cholesterol, interleukin-6 (IL-6)) and whole blood (HbA1c) at immediate labs; for a subsample of patients, triglycerides and HDL-C were later measured from biobank serum for logistical reasons. When we repeated our final analyses with adjustments for the analysis lab, none of the results reported here were changed (data not shown).

### Pre-operative ED biomarkers

We measured each of the 5 ED biomarkers from biobank plasma using enzyme-linked immunosorbent assay (ELISA) kits described as follows: ADMA fast ELISA (CE IVD), SDMA ELISA (CE IVD), sICAM-1, Human ELISA (RUO), sVCAM-1 Human ELISA (RUO) and IMUBIND vWF ELISA (RUO). The analyses were performed according to the manufacturer’s instructions. The only exception was ELISA for vWF, where the dilution factor of 300 was used instead of the recommended dilution factor of 100.

The intra and inter-assay coefficients of variation (CV) were as follows: ADMA, intra-assay CV 7.9% and inter-assay CV 15.9%; SDMA, 12.0% and 18.8%, ICAM-1, 4.4% and 14.5%, VCAM-1, 5.3% and 12.0%, for vWF, 2.4% and 5.5%. To derive a single component explaining the highest variance among all included ED biomarkers, we applied principal component analysis. The standardized regression score resulting from this PCA was saved (‘ED factor’) and used as an additional exposure variable in our main analyses.

### Brain imaging data

Patients underwent 3T MRI during the days before surgery. In Berlin, 3T MagnetomTrio by Siemens was used; in Utrecht, 3T Achieva by Philips was used. Raw images were assessed for indicators of cerebrovascular damage by automated programs and/or trained radiologists. All images (both from Berlin and Utrecht) were processed at a single center. The MRI markers of cerebrovascular damage included volume of WMH (mL), cerebral infarctions, and gray matter cerebral blood flow (arterial spin labeling).

### Postoperative neurocognitive disorders

*Postoperative delirium (POD*) was defined according to the Diagnostic and Statistical Manual of Mental Disorders (DSM-5) criteria. Patients were considered as having POD if any one of the following criteria were met; i) ≥ 2 cumulative points on the nursing delirium screening scale (Nu-DESC), ii) a positive confusion assessment method (CAM) score, iii) a positive CAM for the intensive care unit (CAM-ICU), iv) patient chart review that shows descriptions of delirium (e.g. confused, agitated, drowsy, disorientated, delirious, received antipsychotic therapy for delirium).

*Postoperative cognitive dysfunction (POCD)* was based on six age-sensitive neuropsychological tests of ‘fluid ability’ [48] which were administered before surgery and again at 7 days and 3 months. Four tests were administered on handheld tablet devices from the CANTAB® battery (Verbal Recognition Memory; Paired Associates Learning; Spatial Span; Simple Reaction Test) and two were conventional tests (Trail-Making; Grooved Pegboard). Scores were imputed for patients with missing data on individual tests using the random forest imputation technique. In exceptional cases, where missing data were accompanied by free-text comments such as “impaired concentration”, missing data were replaced by worst-case performance. A non-surgical control group of ≥ 65 year olds was recruited at the study centers [49]. Next, according to Rasmussen criteria [50], POCD was defined for the patient sample as a composite reliable change index (RCI) from all cognitive tests of  > 1.96 across and/or an RCI > 1.96 on any one individual cognitive test.

### Treatment of missing data and outliers

Missing data were imputed as follows: i) for continuous variables including HbA1C (*n* = 95), triglyceride (*n* = 34), total cholesterol (*n* = 94), HDL-C (*n* = 28), missings were replaced as the respective median of the distribution, ii) for categorical variable including hypertension (*n* = 13), diabetes (*n* = 21), stroke (*n* = 17), TIA (*n* = 22), the missing covariate were assumed as absent for the respective patient, and iii) missing data on surgery type (*n* = 17) were assigned as the most frequent surgery type (peripheral surgery). Missing data on MRI variables were not imputed given the high proportion of patients with missing data (60.65%).

The outliers in the concentrations of ED biomarkers were defined and dealt with as follows: i) values which were 1.5 times of interquartile range (IQR) above the third quartile (Q3) were replaced with (1.5 × IQR + Q3) ii) values which was 1.5 times of IQR below the first quartile (Q1) were replaced with (1.5 × IQR– Q1).

### Statistical analyses

Sociodemographic and clinical characteristics were described for the total analysis sample on the outcome POD (*N* = 788), on the analysis sample on the outcome POCD (*n* = 542), and stratified according to the presence or absence of POD and POCD respectively. Group differences between POD and no-POD groups and between POCD and no-POCD groups were assessed using Mann–Whitney U tests for continuous variables and chi-square tests for binary variables. Principal component analysis (PCA) was applied to data on the five ED biomarkers to derive a principal component (PC) of ED. In PCA analysis, a single component with Eigenvalue > 1 was found and the standardized regression score resulting from this PCA was saved and used as an additional exposure variable (‘ED factor’).

The associations among ED biomarkers and those of ED biomarkers with demographic and health-related characteristics were assessed using Spearman correlation and chi-square tests.

Logistic regression analyses determined the associations of each ED marker and the ED factor (as continuous variables and as quartiles) with POD and POCD risk, in three models with hierarchical adjustment for covariates. Separate models were run for each ED marker and the outcomes POD and POCD respectively. The covariates used for adjustments were selected based on their roles as candidate confounders or mediators in the association of ED with POD/POCD. Adjustment was identical for POD and POCD for consistency and comparability of results. Model 1 was adjusted for age (continuous), sex, surgery type (intracranial, intrathoracic, peripheral), and pre-morbid IQ (continuous) as potential confounding factors with potential links both to ED biomarkers and POD/POCD outcomes. For instance, a lower pre-morbid IQ has been associated with poorer lifestyle choices [51, 52] which can cause ED and is potentially also linked to POD/POCD [53, 54]. Such links could lead to spurious associations of ED with POD/POCD in our analysis, thus we controlled for pre-morbid IQ. Model 2 was additionally controlled for BMI (continuous), hypertension (yes/no), diabetes (yes/no), HbA1c (continuous), triglycerides (continuous), total cholesterol (continuous) and HDL-C (continuous), HbA1C (continuous). These vascular risk factors too could function as confounders to the associations under investigation here. In model 3 and model 4, we controlled for potential mediating factors that could link ED with POD/POCD. Specifically, in model 3, TIA (yes/no) and stroke (yes/no) as clinical cerebrovascular disease were further controlled for. Finally, to assess whether the association between ED biomarkers and POD/POCD was mediated by subclinical cerebrovascular damage, in a subset of patients with complete MRI data, MRI markers (WMH volume, cerebral infarction, gray matter cerebral blood flow) model 3 was repeated, and next MRI markers were added to the model (model 4). In a post-hoc analysis, the fully adjusted model on ED biomarkers and POD was repeated with restriction to patients who returned at 3 months. To assess the interdependence of ED biomarkers in association with subsequent POD/POCD development, Model 2 was repeated with inclusion of all 5 ED biomarkers. Also, the fully adjusted model for POCD was controlled for POD to assess a possible mediatory role of POD in the association between ED biomarkers and POCD. Analyses were conducted in SAS 8.3 Update 2 (8.3.2.140), SAS Institute Inc., Cary, NC, USA.

## Results

### Sample characteristics

Of 933 participants, 788 (%) had complete data on POD and ED biomarkers and provided the sample for the analysis of the outcome POD. Of those, 537 (%) returned for a 3-month follow-up and had complete cognitive and ED biomarkers data, and were thus included in our analysis of POCD (Supplementary Fig. 1). No statistically significant differences were found in pre-operative clinical and surgery-related characteristics including age, sex, surgery type, pre-morbid IQ, global cognitive ability (*g*), BMI, hypertension, diabetes, history of stroke and TIA, between patients who were included, and those who were excluded in the analyses of POD and POCD respectively (Supplementary Table 1A & 1B).

Of 788 patients included in the analysis of POD, the median age was 72 years (IQR: 68–76) and 42.5% of the study population was female. Of the 788 patients, 19.7% developed POD. 537 patients were included in the analysis of POCD with a median age of 74 years (IQR: 70–78) with 50% females. Of these 537 patients, 10.1% developed POCD.

Four hundred seventy-two participants had available data on the pre-operative volume of WMH with a median of 2.54 ml (interquartile range, IQR, 1.07–6.00 ml). 478 patients had data on the pre-operative presence or absence of cerebral infarctions (31% with cerebral infarction) and 378 patients had their pre-operative gray matter cerebral blood flow measured with a median of 106.41 ml/100g/min (IQR, 86.20–127.15 ml/100g/min).

### Pre-operative ED biomarkers and pre-operative sociodemographic and clinical characteristics

All five ED biomarkers were strongly positively associated with one another (Spearman’s correlation ranging 0.13 to 0.40; *p* < 0.001, Supplementary Table 2A&2B). In PCA analysis, a single component explained 45% of the variance in the data (factor loadings: VCAM-1 = 0.74; ICAM-1 = 0.72; SDMA = 0.62; vWF = 0.62; ADMA = 0.60).

ADMA was not statistically significantly associated with any sociodemographic and clinical characteristics. SDMA was weakly positively associated with age, and weakly negatively associated with the global ability factor *g* (*p* < 0.01). ICAM-1 was weakly negatively correlated with HDL-C and positively correlated with *g*, HbA1c, and IL-6 (*p* < 0.001). VCAM-1 and vWF were weakly positively correlated with age, BMI, and IL-6 and negatively correlated with *g* (*p* < 0.01). No significant difference was detected for the levels of ED biomarkers once participants were grouped based on sex, hypertension, or diabetes status (Supplementary Table 2A& 2B):

The associations of ED biomarkers with cerebrovascular damage, defined by MRI markers, can be found in Supplementary Tables 3A & 3B. One SD increment in concentrations of ADMA and SDMA was associated with 0.11(ml) and 0.13 (ml) increase in mean volume of WMH (*p* < 0.05). No further associations of ED biomarkers with MRI parameters were detected.

### Pre-operative sociodemographic and clinical characteristics of POD and POCD groups

POD patients were older (*p* < 0.01) and more likely to have intrathoracic/abdominal surgeries (61.7% vs 38.4%, *p* < 0.01) compared with no-POD patients (Table 1).

**Table 1:** Participants' sociodemographic and clinical characteristics at pre-operative assessment (before surgery) according to POD development

Characteristics	Patients with POD (*n* = 156)	Patients without POD (*n* = 632)	*P*-value^1^
Age			
Years (IQR)	74.0 (71.0–76.0)	71.0 (68.0–75.0)	0.006
Sex			
Women (N, %)	73 (46.8)	262 (41.4)	0.19
Surgery type (N, %)			0.008
Intracranial	2 (1.3)	8 (1.3)	
Intrathoracic	97 (61.7)	243 (38.4)	
Peripheral	58 (37.0)	381(60.3)	
BMI (kg/m2)			0.80
Median (IQR)	26.5 (23.6–29.8)	26.7 (24.2–29.4)	
Hypertension (N, %)	104 (66.7)	392 (63.2)	0.42
Diabetes			
Type 1 (N, %)	26 (16.7)	85 (13.7)	0.32
Type 2 (N, %)	42 (27.4)	107 (17.4)	0.006
HbA1C (mmol/mol)			
Median (IQR)	34.9 (30.6–38.2)	34.9 (31.7–39.0)	0.68
Triglyceride (mmol/l)			0.01
Median (IQR)	1.5 (1.1–2.2)	1.5 (1.0–1.9)	
Total cholesterol (mmol/l)			0.01
Median (IQR)	4.60 (3.7–5.2)	4.88 (4.2–5.7)	
HDL cholesterol (mmol/l)			
Median (IQR)	1.1 (0.9–1.50)	1.3(1.1–1.6)	0.01
LDL cholesterol (mmol/l)			
Median (IQR)	2.8 (2.2–3.5)	3.0 (2.3–3.7)	0.03
IL-6 (pg/ml)			
Median (IQR)	2.6 (0.8–6.8)	1.7 (0.0–4.4)	0.001
SDMA			
Median (IQR, SD)	0.7 (0.3, 0.3)	0.7 (0.3, 0.3)	0.23
ADMA			
Median (IQR, SD)	0.8 (0.3, 0.2)	0.7 (0.2, 0.2)	0.02
ICAM-1			
Median (IQR, SD)	658.1 (222.8, 173.3)	649.6 (178.1, 152.3)	0.03
VCAM-1			
Median (IQR, SD)	849.1 (358.8, 398.4)	798.7(305.0, 336.0)	0.007
vWF			
Median (IQR, SD)	832.1 (842.9; 748.7)	861.4 (717.3, 679.6)	0.47
History of transient ischemic attack (N, %)	8 (5.3)	21 (3.4)	0.30
History of stroke (N, %)	10 (5.5)	43 (5.6)	0.63

*Abbreviations*: *ADMA* asymmetric dimethylarginine, *BMI* body mass index, *ICAM-1–1* intercellular adhesion molecule-1, *ICU* intensive care unit, *IL-6* interleukin 6, *IQR* Interquartile range, *POD* post-operative delirium, *SD* Standard deviation, *SDMA* symmetric dimethylarginine, *VCAM-1* vascular cell adhesion molecule-1, *vWF* von Willebrand factor

^1^*P*-value shown for Mann Whitney U test for continuous variables and by chi^2^-square test for categorical variables

POCD patients were significantly older in comparison to those who did not develop POCD (*p* < 0.01, Table 2).

**Table 2:** Participants' sociodemographic and clinical characteristics at pre-operative assessment (before surgery) according to PCOD development

Characteristics^1^	Patients with POCD (*n* = 54)	Patients without POCD (*n* = 483)	*P*-value^1^
Age			
Years (IQR)	74.0 (70.0–78.0)	72.0 (68.0–75.0)	0.002
Sex			
Women (N, %)	27 (50.0)	182 (37.8)	0.10
Surgery type			0.08
Intracranial	2 (3.7)	3 (0.6)	
Intrathoracic	20 (38.9)	199 (42.3)	
Peripheral	30(57.4)	268 (57.0)	
BMI (kg/m^2^)			
Median (IQR)	27.1 (25.0–31.8)	26.5 (24.1–29.1)	0.09
Hypertension (N, %)	36 (66.7)	290 (60.8)	0.40
Diabetes			
Type 1 (N, %)	6 (11.1)	63 (13.5)	0.60
Type 2 (N, %)	11 (20.4)	90 (19.0)	0.80
HbA1C (mmol/mol)			
Median (IQR)	34.9 (30.6–40.0)	34.9 (2.0–38.2)	0.50
Triglyceride (mmol/l)			0.93
Median (IQR)	1.4 (1.1–1.8)	1.4 (1.0–1.8)	
Total cholesterol (mmol/l)			0.19
Median (IQR)	4.7 (4.2–5.4)	5.0 (4.3–5.7)	
HDL (mmol/l)			
Median (IQR)	1.3 (1.1–1.6)	1.3 (1.1–1.6)	0.54
LDL (mmol/l)			
Median (IQR)	2.9 (21–3.3)	3.1 (2.4–3.8)	0.06
IL-6 (pg/ml)			
Median (IQR)	3.2 (0.6–7.3)	1.6 (0.0–4.0)	0.02
SDMA			0.36
Median (IQR)	0.7 (0.3)	0.7 (0.3)	
ADMA			
Median (IQR)	0.7 (0.3)	0.8 (0.2)	0.22
ICAM-1			
Median (IQR)	621.9 (211.7)	644.5 (163.9)	0.41
VCAM-1			
Median (IQR)	757.5 (208.7)	821.6 (309.3)	0.05
vWF			
Median (IQR)	868.6 (694.2)	819.0 (766.0)	0.94
History of stroke (N, %)	3 (5.7)	25 (5.2)	0.90
History of transient ischemic attack (N, %)	2 (3.7)	19 (4.0)	0.92

*Abbreviations*: *ADMA* asymmetric dimethylarginine, *BMI* body mass index, *ICAM-1* intercellular adhesion molecule-1, *ICU* Intensive care unit; IL-6, interleukin 6; IQR, interquartile range, *SDMA* symmetric dimethylarginine, *VCAM-1* vascular cell adhesion molecule-1, *vWF* von Willebrand factor

^1^*P*-value shown for Mann Whitney U test for continuous variables and by chi^2^-square test for categorical variables

### Association of pre-operative ED biomarkers and POD risk

In a model adjusted for age, sex, surgery type and pre-morbid IQ, a significant association was detected between higher concentrations of pre-operative SDMA, ICAM-1, and VCAM-1 and an increased risk of POD (model 1, per one standard deviation (SD) higher concentration of ED biomarkers were associated with a 1.19-fold increased risk; 95% CI: 1.01–1.42 for SDMA, with a 1.22-fold increased risk; 95% CI: 1.03–1.45 for ICAM-1; and with a 1.22-fold increased risk; 95% CI: 1.04–1.45 for VCAM-1).

Furthermore, each SD increment in ED factor was associated with a 1.26-fold increased risk of POD (model 1, 95% CI: 1.04 to 1.52). However, after further adjusting for BMI, hypertension, diabetes, HbA1C, triglycerides, total cholesterol, and HDL-C, no statistically significant associations of levels of ED biomarkers with POD were found (model 2, Table 3). Further adjustment for history of stroke or TIA (model 3, Table 3) as well as repeating model 3 for patients with available data on MRI, and in the next step adding parameters of subclinical cerebrovascular damage from brain imaging to the model, produced similar results (model 3&4, Table 4).

**Table 3:** The association between pre-operative ED biomarkers with POD risk in study participants (*n* = 788)

	Quartiles				*P*-Trend	Continuous Variable	
	1	2	3	4		OR	*P*-value
						per one-SD increment	
ADMA							
Cut point (μmol/l)	≤ 0.65	0.66–0.76	0.77–0.90	≥ 0.91			
N with POD/ N total	36/ 197	27/ 197	50/ 197	44/ 198			
Model 1 OR (95% CI)	Ref	0.68 (0.39 to 1.19)	1.51 (0.92 to 2.50)	1.14 (0.68 to 1.91)	0.15	1.17 (0.92 to 1.41)	0.06
Model 2 OR (95% CI)	Ref	0.65 (0.36 to 1.17)	1.35 (0.79 to 2.29)	1.04 (0.61 to 1.79)	0.34	1.12 (0.93 to 1.36)	0.11
Model 3 OR (95% CI)	Ref	0.64 (0.35 to 1.15)	1.32 (0.77 to 2.24)	1.04 (0.61 to 1.79)	0.09	1.13 (0.93 to 1.36)	0.21
SDMA							
Cut point (μmol/l)	≤ 0.60	0.61–0.74	0.75–0.90	≥ 0.91			
N with POD/ N total	40/ 197	36/ 197	33/ 198	48/ 197			
Model 1 OR (95% CI)	Ref	0.77 (0.46 to 1.30)	0.66 (0.39 to 1.13)	1.03 (0.63 to 1.70)	0.97	1.19 (1.01 to 1.42)	0.04
Model 2 OR (95% CI)	Ref	0.68 (0.39 to 1.19)	0.60 (0.34 to 1.06)	0.84 (0.49 to 1.45)	0.54	1.14 (0.96 to 1.35)	0.11
Model 3 OR (95% CI)	Ref	0.71 (0.40 to 1.24)	0.54 (0.30 to 0.96)	0.87 (0.51 to 1.50)	0.27	1.14 (0.96 to 1.35)	0.12
ICAM-1							
Cut point (ng/ml)	≤ 566	567–647	648–743	≥ 744			
N with POD/ N total	40/ 197	34/ 197	33/ 198	48/ 197			
Model 1 OR (95% CI)	Ref	1.09 (0.64 to 1.85)	1.27 (0.75 to 1.14)	1.37 (0.81 to 2.30)	0.18	1.22 (1.03 to 1.45)	0.01
Model 2 OR (95% CI)	Ref	1.21 (0.63 to 1.96)	1.16 (0.66 to 2.04)	1.21 (0.69 to 2.13)	0.49	1.16 (0.96 to 1.40)	0.09
Model 3 OR (95% CI)	Ref	1.16 (0.63 to 1.96)	1.17 (0.66 to 2.06)	1.23 (0.70 to 2.17)	0.89	1.17 (0.96 to 1.41)	0.10
VCAM-1							
Cut point (ng/ml)	≤ 681	682–807	800–986	≥ 987			
N with POD/ N total	32 /197	36/ 197	42/ 198	47/ 197			
Model 1 OR (95% CI)	Ref	0.98 (0.57 to 1.68)	1.17 (0.69 to 1.98)	1.47 (0.88 to 2.46)	0.09	1.22 (1.04 to 1.45)	0.01
Model 2 OR (95% CI)	Ref	0.72 (0.40 to 1.28)	0.93 (0.53 to 1.64)	1.10 (0.63 to 1.93)	0.46	1.15 (0.96 to 1.38)	0.10
Model 3 OR (95% CI)	Ref	0.70 (0.39 to 1.25)	0.94 (0.53 to 1.66)	1.11 (0.63 to 1.94)	0.42	1.16 (0.97 to 1.39)	0.10
vWF							
Cut point (mU/ml)	≤ 569	570–853	854–1308	≥ 1309			
N with POD/ N total	46/ 197	45 / 197	29/ 198	46/ 197			
Model 1 OR (95% CI)	Ref	1.16 (0.70 to 1.93)	1.04 (0.60 to 1.79)	0.97 (0.56 to 1.68)	0.93	1.01 (0.84 to 1.21)	0.87
Model 2 OR (95% CI)	Ref	1.06 (0.61 to 1.83)	0.59 (0.33 to 1.05)	0.98 (0.57 to 1.70)	0.50	0.95 (0.77 to 1.16)	0.66
Model 3 OR (95% CI)	Ref	1.05 (0.61 to 1.82)	0.59 (0.33 to 1.04)	0.99 (0.57 to 1.71)	0.16	0.92 (0.78 to 1.17)	0.70
ED factor							
N with POD/ N total	32/ 197	36/ 197	37/ 197	51/ 197			
Model 1 OR (95% CI)	Ref	1.05 (0.61 to 1.80)	1.10 (0.64 to 1.88)	1.62 (0.97 to 2.70)	0.19	1.26 (1.04 to 1.52)	0.01
Model 2 OR (95% CI)	Ref	0.91 (0.51 to 1.64)	1.04 (0.59 to 1.85)	1.27 (0.73 to 2.21)	0.66	1.51 (0.93 to 1.45)	0.18
Model 3 OR (95% CI)	Ref	0.93 (0.52 to 1.57)	1.04 (0.59 to 1.85)	1.30 (0.74 to 2.27)	0.63	1.15 (0.93 to 1.42)	0.17

Results are shown for logistic regression analyses with outcome POD. The *p*-value for trend (2-sided) is based on the Wald chi^2^ statistic. OR per 1-SD increment refers to the change in risk of POD per 1 SD increment in ED biomarker. For instance, an OR 1.5 would mean that for each 1 SD increment in ED biomarker exposure, the risk of the outcome is 1.5-fold. OR in quartiles 2, 3 and 4 refer to the risk of POD relative to quartile 1 as reference quartile

Following the limitation of the outliers to the upper or lower control limits, for ADMA (*n* = 13), SDAM (*n* = 19), ICAM-1 (*n* = 28), VCAM-1 (*n* = 35), vWF (37), results remained largely unchanged

Model 1: adjusted for age (continuous), sex, surgery type (intracranial: 1.3% in both patients with and without POD, intrathoracic: 63.8% in patients with POD vs 31.5% in patients without POD. peripheral: 34.9% in patients with POD vs 59.5% in patients without POD), pre-morbid IQ (continuous)

Model 2: Model 1 + BMI (continuous), hypertension (yes/no), diabetes (yes/no), HbA1C (continuous), triglyceride (continuous), total cholesterol (continuous), HDL-C (continuous)

Model 3: Model 2 + stroke (yes/no), TIA (yes/no)

Addition of quadratic terms to the respective Model 2 led to the following results: ADMA^2^, *p* = 0.58; SDMA^2^, *p* = 0.40; VCAM-1^2^, *p* = 0.28; ICAM-1^2^, *p* = 0.76; vWF, *p* = 0.34

*Abbreviations*: *ADMA* asymmetric dimethylarginine, *CI* confidence interval, *ICAM-1* intercellular adhesion molecule, *OR* odds ratio, *POD* post-operative delirium, *SDMA* symmetric dimethylarginine, *TIA* transient ischemic attack, *VCAM-1* vascular cell adhesion molecule-1, *vWF*von Willebrand factor

**Table 4:** The associations between pre-operative ED biomarkers with POD risk in study participants with available data on MRI (*n* = 310)

	Quartiles				*P*-Trend	Continuous Variable	
	1	2	3	4		OR	*P*-value
						per one-SD increment	
ADMA							
Cut point (μmol/l)	≤ 0.65	0.66–0.76	0.77–0.90	≥ 0.91			
N with POD/ N total	12/ 77	5/ 77	16/ 79	12/ 77			
OR Model 3(95% CI)	Ref	0.45 (0.13 to 1.56)	1.32 (0.47 to 3.69)	0.91 (0.32 to 2.58)	0.29	1:04 (0.72 to 1.49)	0.40
OR Model 4 (95% CI)	Ref	0.45 (0.13 to 1.56)	1.41 (0.51 to 3.90)	0.89 (0.31 to 2.52)	0.35	1.02 (0.70 to 1.48)	0.75
SDMA							
Cut point (μmol/l)	≤ 0.60	0.61–0.74	0.75–0.90	≥ 0.91			
N with POD/ N total	13/ 77	10/ 78	11/ 78	11/ 77			
OR Model 3(95% CI)	Ref	0.88 (0.31 to 2.38)	0.79 (0.27 to 2.27)	0.66 (0.22 to 1.93)	0.90	1.19 (0.89 to 1.60)	0.47
OR Model 4 (95% CI)	Ref	0.66 (0.30 to 2.43)	0.87 (0.27 to 2.68)	0.66 (0.23 to 1.95)	0.78	1.19 (0.88 to 1.59)	0.23
ICAM-1							
Cut point (ng/ml)	≤ 566	567–647	648–743	≥ 744			
N with POD/ N total	10/ 77	10/ 78	11/ 78	14/ 77			
OR Model 3(95% CI)	Ref	1.07 (0.35 to 3.22)	1.29 (0.41 to 4.02)	1.30 (0.43 to 3.92)	0.95	1.02 (0.71 to 1.45)	0.90
OR Model 4 (95% CI)	Ref	1.13 (0.37 to 3.42)	1.26 (0.40 to 3.93)	1.24 (0.41 to 3.78)	0.97	0.99 (0.62 to 1.49)	0.99
VCAM-1							
Cut point (ng/ml)	≤ 681	682–807	800–986	≥ 987			
N with POD/ N total	9/ 77	12/ 78	8/ 78	16/ 77			
OR Model 3(95% CI)	Ref	0.91 (0.31 to 2.61)	0.53 (0.16 to 1.73)	1.24 (0.43 to 3.59)	0.44	0.99 (0.68 to 1.43)	0.97
OR Model 4 (95% CI)	Ref	0.97 (0.33 to 2.79)	0.54 (0.17 to 1.79)	1.23 (0.43to 3.51)	0.50	0.97 (0.67 to 1.41)	0.88
vWF							
Cut point (mU/ml)	≤ 569	570–853	854–1308	≥ 1309			
N with POD/ N total	10/ 77	18 / 78	8/ 78	9/ 77			
OR Model 3(95% CI)	Ref	1.50 (0.56 to 4.03)	0.69 (0.23 to 2.06)	0.56 (0.18 to 1.70)	0.26	0.69 (0.44 to 1.08)	0.08
OR Model 4 (95% CI)	Ref	1.53 (0.56 to 4.17)	0.66 (0.22 to 2.01)	0.55 (0.17 to 1.70)	0.23	0.68 (0.43 to 1.08)	0.10
ED factor							
OR Model 3(95% CI)	Ref	1.51 (0.56 to 4.09)	1.04 (0.33 to 3.07)	0.84 (0.27 to 2.45)	0.81	0.97 (0.65 to 1.43)	0.91
OR Model 4 (95% CI	Ref	1.53 (0.56 to 4.17)	1.02 (0.34 to 3.04)	0.83 (0.28 to 2.45)	0.76	0.96 (0.64 to 1.45)	0.87

Results are shown for logistic regression analyses with outcome POD. The *p*-value for trend (2-sided) is based on the Wald chi^2^ statistic. OR per 1-SD increment refers to the change in risk of POD per 1 SD increment in ED biomarker. For instance, an OR 1.5 would mean that for each 1 SD increment in ED biomarker exposure, the risk of the outcome is 1.5-fold. OR in quartiles 2, 3 and 4 refer to the risk of POD relative to quartile 1 as reference quartile

Model 3 adjusted for age (continuous), sex, surgery type (intracranial: 3.7% in patients with POD vs 0.6% without POD, intrathoracic: 39.0% in patients with POD vs 38.1% without POD. peripheral: 57.4% in patients with POD vs 51.1% without POD), pre-morbid IQ (continuous), triglyceride (continuous), HDL-C (continuous), total cholesterol (continuous), BMI (continuous), hypertension (yes/no), diabetes (yes/no), HbA_1_C (continuous), TIA (yes/no), stroke (yes/no)

Model 4 adjusted for Model 3 + gm-c blood flow (continuous), volume of WMH (continuous), cerebral infarctions (yes/no)

*Abbreviations*: *ADMA* asymmetric dimethylarginine, *CI* confidence interval, *gm-c* Gray matter cerebral, *ICAM-1–1* intercellular adhesion molecule, *MRI* magnetic resonance imaging, *OR* odds ratio, *POD* post-operative delirium, *SDMA* symmetric dimethylarginine, *TIA* transient ischemic attack, *VCAM-1* vascular cell adhesion molecule-1, *vWF* von Willebrand factor, *WMH* white matter hyperintensities

When restricting the analysis to POD patients who returned at 3 months follow-up (*n* = 537), the results remain similar but no longer significant in model 1 for ICAM-1, and VCAM-1 (data not shown). Results stayed similar for model 2 and model 3. Additionally, when repeating in all models with the inclusion of all ED biomarkers in the model, the results remained mostly similar (data not shown).

### Association of pre-operative ED biomarkers with POCD risk

In the model adjusted for age, sex, surgery type and pre-morbid IQ, higher VCAM-1 and a higher ED factor were each statistically significantly associated with a reduced POCD risk (model 1, one SD increase in VCAM-1 levels was associated with a 0.64-fold POCD risk; OR, 0.64 95% CI: 0.43 to 0.95, and one SD increase in ED factor was associated with a 0.82-fold POCD risk; OR, 0.82 95% CI: 0.69 to 0.98). For VCAM-1, the association was also statistically significant in quartile analyses, persisted after adjusting for BMI, hypertension, diabetes, HbA1C, triglyceride, total cholesterol, HDL-C, stroke and TIA (model 2&3), and after repeating model 3 for patients with available data on subclinical cerebrovascular damage from brain imaging as well as after further adjustments for cerebrovascular damage on MRI (model 3&4, Table 6). In the fully adjusted model, the risk of POCD in the highest VCAM-1 quartile was 0.17-fold (95% CI 0.03 to 0.84) compared with the lowest quartile (Table 6). No other significant associations were found for ICAM-1, ADMA, SDMA and vWF, and POCD risk (Tables 5 and 6). The results remained similar after the inclusion of all ED biomarkers or after additionally controlling for POD (data not shown).

**Table 5:** The associations between pre-operative ED biomarkers with POCD risk in study participants (*n* = 537)

	Quartiles				*P*-Trend	Continuous Variable	
	1	2	3	4		OR	*P*-value
						per one-SD incrementease	
ADMA							
Cut point (μmol/l)	≤ 0.64	0.65–0.75	0.76–0.89	≥ 1.54			
N with POCD/ N total	18/ 134	15/135	8/134	13/134			
Model 1 OR (95% CI)	Ref	0.71(0.33 to 1.51)	0.37 (0.15to 0.90)	0.66 (0.30 to 1.43)	0.14	0.81 (0.60 to 1.10)	0.19
Model 2 OR (95% CI)	Ref	0.79 (0.35 to 1.78)	0.44 (0.17 to 1.13)	0.74 (0.32 to 1.69)	0.30	0.89 (0.65 to 1.21)	0.47
Model 3 OR (95% CI)	Ref	0.79 (0.35 to 1.79)	0.44 (0.17 to 1.13)	0.73 (0.31 to 1.68)	0.29	0.88 (0.65 to 1.21)	0.45
SDMA							
Cut point (μmol/l)	≤ 0.60	0.61–0.74	0.75–0.89	≥ 4.10			
N with POCD/ N total	17/134	11/135	14/135	12/134			
Model 1 OR (95% CI)	Ref	0.55 (0.24 to 1.26)	0.64 (0.29 to 1.41)	0.54 (0.24 to 1.22)	0.18	0.74 (0.50 to 1.09)	0.13
Model 2 OR (95% CI)	Ref	0.54 (0.23 to 1.30)	0.70 (0.30 to 1.62)	050 (0.20 to 1.23)	0.20	0.78 (0.52 to 1.16)	0.22
Model 3 OR (95% CI)	Ref	0.55 (0.23 to 1.31)	0.71 (0.30 to 1.64)	0.51 (0.21 to 1.25)	0.22	0.78 (0.52 to 1.17)	0.23
ICAM-1							
Cut point (ng/ml)	≤ 558	558–641	642–727	≥ 1317			
N with POCD/ N total	17/134	14/134	9/135	14/134			
Model 1 OR (95% CI)	Ref	0.81 (0.37 to 1.75)	0.55 (0.23 to 1.31)	0.87(0.40 to 1.88)	0.60	0.89 (0.66 to 1.20)	0.46
Model 2 OR (95% CI)	Ref	0.97 (0.42 to 2.26)	0.65 (0.25 to 1.66)	0.96 (0.40 to 2.28)	0.74	0.88 (0.63 to 1.24)	0.43
Model 3 OR (95% CI)	Ref	0.97 (0.42 to 2.27)	0.65 (0.25 to 1.68)	0.95 (0.40 to 2.28)	0.73	0.87 (0.63 to 1.21)	0.43
VCAM-1							
Cut point (ng/ml)	≤ 673	674–807	808–980	≥ 3854			
N with POCD/ N total	19/134	17/135	10/135	8/134			
Model 1 OR (95% CI)	Ref	0.78 (0.38 to 1.61)	0.39 (0.16 to 0.91)	0.35 (0.14 to 0.85)	0.01	0.64 (0.43 to 0.95)	0.02
Model 2 OR (95% CI)	Ref	0.80 (0.37 to 1.73)	0.35 (0.13 to 0.88)	0.23 (0.08 to 0.63)	0.01	0.55 (0.35 to 0.86)	0.007
Model 3 OR (95% CI)	Ref	0.81 (0.37 to 1.85)	0.34 (0.13 to 0.88)	0.23 (0.08 to 0.63)	0.001	0.55 (0.35 to 0.86)	0.01
VWF							
Cut point (mU/ml)	≤ 534	535–824	825–1294	≥ 5830			
N with POCD/ N total	14/134	11/134	17/135	12/134			
Model 1 OR (95% CI)	Ref	0.71 (0.30 to 1.63)	1.30 (0.60 to 2.85)	0.73 (0.31 to 1.68)	0.81	0.87 (0.63 to 1.20)	0.40
Model 2 OR (95% CI)	Ref	0.80 (0.31 to 2.01)	1.45 (0.62 to 3.40)	0.72 (0.28 to 1.81)	0.80	0.82 (0.58 to 1.16)	0.27
Model 3 OR (95% CI	Ref	0.78 (0.31 to 1.95)	1,44 (0.61 to 3.37)	0.70 (0.28 to 1.78)	0.78	0.81 (0.57 to 1.16)	0.26
ED factor							
N with POCD/ N total	14/ 134	13/134	16/135	11/134			
Model 1 OR (95% CI)	Ref	0.89 (0.39 to 2.02)	1.11 (0.51 to 2.43)	0.71 (0.30 to 1.65)	0.74	0.82 (0.69 to 0.98)	0.01
Model 2 OR (95% CI)	Ref	1.18 (0.44 to 2.80)	1.44 (0.60 to 3.47)	1.66 (0.25 to 1.74)	0.66	0.82 (0.66 to 1.02)	0.18
Model 3 OR (95% CI)	Ref	1.12 (0.44 to 2.81)	1.44 (0.60 to 3.48)	0.65 (0.25 to 1.73)	0.38	0.82 (0.66 to 1.02)	0.07

Results are shown for logistic regression analyses with outcome POCD. The *p*-value for trend (2-sided) is based on the Wald chi^2^ statistic. OR per 1-SD increment refers to the change in risk of POCD per 1 SD increment in ED biomarker. For instance, an OR 1.5 would mean that for each 1 SD increment in ED biomarker exposure, the risk of the outcome is 1.5-fold. OR in quartiles 2, 3 and 4 refer to the risk of POCD relative to quartile 1 as reference quartile

Following the limitation of the outliers to the upper or lower control limits for ADMA (*n* = 8), SDAM (*n* = 16), SICAM-1 (*n* = 15), SVCAM-1 (*n* = 21), vWF (11), results remained mainly unchanged

Model 1: adjusted for age (continuous), sex, surgery type (intracranial: 3.7% in patients with POCD vs 0.6% without POCD, intrathoracic: 39.0% in patients with POCD vs 38.1% without POCD, peripheral: 57.4% in patients with POCD vs 51.1% without POCD), pre-morbid IQ (continuous)

Model 2: Model 1 +  BMI (continuous), hypertension (yes/no), diabetes (yes/no), HbA1C (continuous), triglyceride (continuous), total cholesterol (continuous), HDL-C (continuous)

Model 3: Model 2 + TIA (yes/no), stroke (yes/no)

Addition of quadratic terms to the respective Model 2 led to the following results: ADMA^2^, *p* = 0.21; SDMA^2^, *p* = 0.93; VCAM-12, *p* = 0.64; sICAM-12, *p* = 0.57; vWF, *p* = 0.40

*Abbreviations*: *ADMA* asymmetric dimethylarginine, *CI* confidence interval, *ICAM-1–1* intercellular adhesion molecule, *OR* odds ratio, *POCD* Post-operative cognitive dysfunction, *SDMA* symmetric dimethylarginine, *TIA* transient ischemic attack, *VCAM-1* vascular cell adhesion molecule-1, *vWF* von Willebrand factor

**Table 6:** The associations between pre-operative ED biomarkers with POCD risk in study participants with available data on MRI (*n* = 252)

	Quartiles				*P*-Trend	Continuous Variable	
	1	2	3	4		OR	*P*-value
						per one-SD incrementease	
ADMA							
Cut point (μmol/l)	≤ 0.64	0.65–0.75	0.76–0.89	≥ 1.54			
N with POCD/ N total	5/63	10/62	5/64	5/63			
OR Model 3(95% CI)	Ref	1.60 (0.44 to 5.81)	0.74 (0.17 to 3.18)	0.72 (0.17 to 3.09)	0.60	0.90 (0.55 to 1.49)	0.70
OR Model 4 (95% CI)	Ref	1.11 (0.26 to 4.73)	0.65 (0.12 to 3.45)	0.67 (0.14 to 3.22)	0.45	0.99 (0.45 to 1.61)	0.81
SDMA							
Cut point (μmol/l)	≤ 0.60	0.61–0.74	0.75–0.89	≥ 4.10			
N with POCD/ N total	6/63	4/63	9/64	6/62			
OR Model 3(95% CI)	Ref	0.64 (0.14 to 2.78)	0.83 (0.21 to 3.21)	0.80 (0.19 to 3.21)	0.95	0.80 (0.46 to 1.40)	0.44
OR Model 4 (95% CI)	Ref	0.91 (0.20 to 4.07)	1.20 (0.26 to 5.42)	0.93 (0.20 to 4.33)	0.98	0.83 (0.50 to 1.37)	0.47
ICAM-1							
Cut point (ng/ml)	≤ 558	558–641	642–727	≥ 1317			
N with POCD/ N total	6/63	7/63	6/63	6/63			
OR Model 3(95% CI)	Ref	1.42 (0.37 to 5.44)	1.07 (0.27 to 4.71)	1.20 (0.30 to 4.71	0.95	1.02 (0.60 to 1.92)	0.90
OR Model 4 (95% CI)	Ref	2.46 (0.63 to 8.07)	1.23 (0.25 to 5.98)	1.20 (0.23 to 6.14)	0.98	1.08 (0.60 to 1.92)	0.78
VCAM-1							
Cut point (ng/ml)	≤ 673	674–807	808–980	≥ 3854			
N with POCD/ N total	7/63	8/63	7/63	3/63			
OR Model 3(95% CI)	Ref	1.00 (0.28 to 3.53)	0.64 (0.17 to 2.38)	0.27 (0.43 to 0.98)	0.19	0.47 (0.22 to 1.01)	0.05
OR Model 4 (95% CI)	Ref	0.74 (0.20 to 2.69)	0.55 (0.14 to 2.16)	0.17 (0.03 to 0.84)	0.02	0.50 (0.24 to 1.02)	0.05
vWF							
Cut point (mU/ml)	≤ 534	535–824	825–1294	≥ 5830			
N with POCD/ N total	5/63	4/63	9/63	7/63			
OR Model 3(95% CI)	Ref	0.39 (0.06 to 2.24)	1.61 (0.45 to 5.70)	0.98 (0.25 to 3.77)	0.38	1.02 (0.63 to 1.64)	0.91
OR Model 4 (95% CI)	Ref	0.64 (0.12 to 3.38)	2.06 (0.51 to 8.32)	1.25 (0.27 to 5.82)	0.47	1.06 (0.56 to 2.02)	0.83
ED factor							
OR Model 3(95% CI)	Ref	1.01 (0.13 to 5.28)	1.95 (0.36 to 9.58)	0.74 (0.16 to 3.82)	0.57	0.90 (0.63 to 1.29)	0.62
OR Model 4 (95% CI)	Ref	1.31 (0.27 to 6.38)	2.73 (0.64 to 11.68)	0.97 (0.21 to 4.52)	0.41	0.90 (0.63 to 1.29)	0.57

Results are shown for logistic regression analyses with outcome POCD. The *p*-value for trend (2-sided) is based on the Wald chi^2^ statistic. OR per 1-SD increment refers to the change in risk of POCD per 1 SD increment in ED biomarker. For instance, an OR 1.5 would mean that for each 1 SD increment in ED biomarker exposure, the risk of the outcome is 1.5-fold. OR in quartiles 2, 3 and 4 refer to the risk of POCD relative to quartile 1 as reference quartile

Model 3 adjusted for age (continuous), sex, surgery type (intracranial: 3.7% in patients with POD vs 0.6% without POD, intrathoracic: 39.0% in patients with POD vs 38.1% in patients without POD. peripheral: 57.4% in patients with POD vs 51.1% in patients without POD), pre-morbid IQ (continuous), triglyceride (continuous), HDL-C (continuous), total cholesterol (continuous), BMI (continuous), HbA_1_C (continuous), hypertension (yes/no), diabetes (yes/no), TIA (yes/no), stroke (yes/no)

Model 4 adjusted for Model 3 + gm-c blood flow (continuous), volume of WMH (continuous), and cerebral infarctions (yes/no)

*Abbreviations*: *ADMA* asymmetric dimethylarginine, *CI* confidence interval, *gm-c* gray matter cerebral, *ICAM-1–1* intercellular adhesion molecule, *MRI* magnetic resonance imaging, *OR* odds ratio,*POCD* Post-operative cognitive dysfunction, *SDMA* symmetric dimethylarginine, *TIA* transient ischemic attack, *VCAM-1* vascular cell adhesion molecule-1, *vWF* von Willebrand factor, *WMH *white matter hyperintensity

## Discussion

### Principal findings

In the first study to assess ED in the context of POD and POCD with consideration of neuroimaging data, we found that higher pre-operative concentrations of SDMA, ICAM-1, VCAM-1 and higher scores on a composite ED factor were each associated with an increased risk of developing POD.  However, we detected a potential confounding role for BMI, hypertension, diabetes, HbA1C, triglyceride, total cholesterol and/or HDL-C in those associations. Individuals with higher VCAM-1 had a lower risk of developing POCD, and this association remained statistically significant after adjustments for all covariates considered in our analyses including cerebrovascular pathology evidenced on MRI.

### Pre-operative ED biomarkers and POD

A number of non-modifiable risk factors for POD have been identified, including age, pre-operative neurocognitive disorders, hypertension, and diabetes [55]. Other, modifiable, risk factors include peri-operative pharmacological and anaesthesiologic treatment, invasiveness and duration of surgical measures as well as suboptimal hydration and temperature homeostasis [56]. In addition to these factors, a role could be considered for systemic ED in causing an increased risk for POD, given the large endothelial surface of the brain and accordingly its likely vulnerability to systemic ED [26–28]. If this was to be the case, then patients' risk of POD could be reduced via controlling ED, though this – as it requires lifestyle changes – would certainly prove difficult in the immediate pre-surgical setting.

ED and POD risk have rarely been assessed in the past. In one cohort of 118 older surgery patients in Germany, no association was found between systematic ED, measured by several biomarkers including ICAM-1-1 and VCAM-1-1, and POD risk [37]. Similarly, a cohort of 300 surgical patients in the US reported, no difference in pre-operative vascular endothelial growth factor (VEGF; a protein involved in angiogenesis) between POD patients and controls [36]. Given the smaller sample size in these two studies, it was suggested that the results might suffer from low power for detecting true associations. Another study of 117 older surgical patients in Japan reported an association of higher pre-operative systemic ED, measured by p-selectin, and an increased POD risk [38]. Importantly, these studies had applied only statistical adjustments for a few selected potential confounding (i.e., age, sex, surgery type, pre-operative cognition, vascular comorbidity, and apolipoprotein E genotype [36], and propensity scoring adjustments for confounders [37]. Here, we controlled our analyses for a large set of potential confounding variables, demonstrating the independence of associations of ED with POD from age, sex, surgery type and pre-morbid IQ, and a dependence on the vascular risk factors BMI, hypertension, diabetes, HbA1c, triglycerides, total cholesterol and/or HDL-C.

 An in-depth evaluation of this finding is needed in future studies. Short of a causal relationship and possible interventions, knowledge of risk factors (irrespective of confounding) is also useful, because it can screen patients to identify those who require further pre-operative evaluation and adjustments in the management of perioperative monitoring. Here, we have shown that measuring pre-operative ED biomarker concentrations could possibly help towards that goal.

We additionally found an association between higher concentrations of ADMA and SDMA with increased WMH volume, which is in line with the research literature associating ED with cerebrovascular damage [29, 41–43]. No further associations of ED biomarkers with the brain imaging parameters including WMH, cerebral infarction or cerebral blood flow were identified, however, and additional adjustment for these brain imaging parameters did not alter the statistically non-significant results on the associations of ED biomarkers with POD.

### Pre-operative ED biomarkers and POCD

POCD shares some risk factors, such as advanced age, with POD [57] but we here have provided evidence for the two conditions as distinct entities with distinct risk factors. Specifically, we observed an inverse association of VCAM-1 with POCD risk at 3 months (which was independent of all considered covariates including subclinical cerebrovascular disease which we suggested could function as a mediator). In combination, this pattern of results could speak to differential mechanisms involved in POD and POCD development. Consistent with this, we also found no association of POD with POCD in our cohort which contrasts with results from a recent meta-analysis of 18 studies which concluded that patients with POD are at increased POCD risk [58].

Previous studies frequently reported higher concentrations of VCAM-1 in patients with dementia as compared with unimpaired controls [29, 30, 59].

Further, inflammation has been proposed as an involved mechanism in the pathogenesis of POCD [60]. On that basis, and with VCAM-1 as a promoter of leukocyte migration across the endothelium to sites of inflammation [61], the observed inverse association between VCAM-1 and POCD development in the current study is particularly surprising and may well be a chance finding or could be driven by residual confounding. Thus, caution is warranted in the interpretation this finding in particular.

### Study strengths and limitations

We used a large cohort with detailed cognitive assessment as well as brain imaging data to investigate the association of pre-operative concentrations of ED with the risk of post-operative neurocognitive disorders. We characterized systemic ED using 5 biomarkers and assessed the interdependence in their relationships with POD/POCD. We adjusted for a range of potential confounding factors and further assessed the mediatory role of pre-operative clinical and subclinical cerebrovascular damage in the association between ED biomarkers and POD/POCD risk. The validity of our POD/POCD measurement was indicated by incidences of these conditions that were consistent with incidence reports in the literature. Yet, our study was accompanied by some limitations. We used a sample of patients who underwent diverse surgical procedures with different anesthetic techniques. Surgical factors such as those are strong risk factors for POD/POCD and despite controlling for “surgery type” in our analyses, a contribution of such factors to our findings may be possible. POD definition did not consider subsyndromal POD. Thus, inclusion of patiens with subsyndromal POD in the “no POD” group will have weakened any biomarker associations with POD. Because we entered correlated variables into the models, multicollinearity may have impacted our results by biasing exposure-outcome associations and reducing precision. In fact, the change in findings between model 1 and model 2 on POD may have stemmed from this. Based on our findings, we are also unable to tease out the roles of specific confounding factors in the association of ED biomarkers with POD/POCD. For instance, the change from model 1 to model 2 could stem from confounding by a lower HDL-C but also from a higher BMI (leading both to higher ED and POD/POCD risk respectively); the step from model 2 to model 3 had been pre-planned to reflect mediation by the state of the cerebrovasculature (clinically as TIA/stroke or subclinically on brain imaging) but due to the loss of statistical significance between model 1 and model 2, model 3 did not add any further information. Brain imaging data was available for only a subset of patients. Thus, analyses of POCD and those involving brain imaging data were affected by a reduced statistical power as compared with the analyses of the full sample for the outcome POD, and by the possibility for an influence of selection bias. However, when we repeated our analyses of POD with restriction to patients who also had data on POCD, or to those with brain imaging data respectively, our results did not change substantially, indicating that reduced power but not selection bias could be a contributing factor here. We linked a total of five exposures with two outcomes which resulted in a relatively large number of statistical analyses and risk of type I error. If we applied a Bonferroni correction with resulting *p*-value of 0.005 for statistical significance (5 exposures, 2 outcomes), the results on VCAM-1 and POCD would survive this correction though the results for ED biomarkers and POD would not. This is to be considered in the interpretation of our results.

## Conclusion

We did not find evidence for concentrations of biomarkers of systemic ED as risk factors for POD over and above their roles as correlates of vascular risk factors. Our analysis surprisingly showed an inverse association between biomarkers of systemic ED and POCD at 3 months after surgery, which was independent of all considered covariates and requires repeat assessment in other cohorts. Our findings do support the notion that POD and POCD are separate entities with distinct etiology and epidemiology.

## Supplementary Information

Supplementary Material 1.

## Data Availability

The datasets used and/or analysed for this study available from the corresponding author on reasonable request.

## References

[1] Weiser TG, Haynes AB, Molina G, et al. Estimate of the global volume of surgery in 2012: an assessment supporting improved health outcomes. Lancet. 2015;385(Suppl 2):S11. 10.1016/s0140-6736(15)60806-6. 26313057 10.1016/S0140-6736(15)60806-6

[2] Mattison MLP. Delirium. Ann Intern Med. 2020;173(7):Itc49-itc64. 10.7326/aitc202010060. 33017552 10.7326/AITC202010060

[3] Janjua MS, Spurling BC, Arthur ME. Postoperative delirium. In: StatPearls. Treasure Island: StatPearls Publishing Copyright © 2022, StatPearls Publishing LLC.; 2022. 30521252

[4] Ho MH, Nealon J, Igwe E, et al. Postoperative delirium in older patients: a systematic review of assessment and incidence of postoperative delirium. Worldviews Evid Based Nurs. 2021;18(5):290–301. 10.1111/wvn.12536. 34482593 10.1111/wvn.12536

[5] Liu J, Huang K, Zhu B, et al. Neuropsychological tests in post-operative cognitive dysfunction: methods and applications. Front Psychol. 2021;12:684307. 10.3389/fpsyg.2021.684307. 34149572 10.3389/fpsyg.2021.684307 PMC8212929

[6] Borchers F, Spies CD, Feinkohl I, et al. Methodology of measuring postoperative cognitive dysfunction: a systematic review. Br J Anaesth. 2021;126(6):1119–27. 10.1016/j.bja.2021.01.035. 33820655 10.1016/j.bja.2021.01.035

[7] Coburn M, Fahlenkamp A, Zoremba N, Schaelte G. Postoperative cognitive dysfunction: incidence and prophylaxis. Anaesthesist. 2010;59(2):177–84. 10.1007/s00101-009-1657-2. quiz 85. 20084351 10.1007/s00101-009-1657-2

[8] Witlox J, Eurelings LS, de Jonghe JF, Kalisvaart KJ, Eikelenboom P, van Gool WA. Delirium in elderly patients and the risk of postdischarge mortality, institutionalization, and dementia: a meta-analysis. JAMA. 2010;304(4):443–51. 10.1001/jama.2010.1013. 20664045 10.1001/jama.2010.1013

[9] Robinson TN, Raeburn CD, Tran ZV, Angles EM, Brenner LA, Moss M. Postoperative delirium in the elderly: risk factors and outcomes. Ann Surg. 2009;249(1):173–8. 10.1097/SLA.0b013e31818e4776. 19106695 10.1097/SLA.0b013e31818e4776

[10] Ruggiero C, Bonamassa L, Pelini L, et al. Early post-surgical cognitive dysfunction is a risk factor for mortality among hip fracture hospitalized older persons. Osteoporos Int. 2017;28(2):667–75. 10.1007/s00198-016-3784-3. 27717957 10.1007/s00198-016-3784-3

[11] Newman MF, Grocott HP, Mathew JP, et al. Report of the substudy assessing the impact of neurocognitive function on quality of life 5 years after cardiac surgery. Stroke. 2001;32(12):2874–81. 10.1161/hs1201.099803. 11739990 10.1161/hs1201.099803

[12] Steinmetz J, Christensen KB, Lund T, Lohse N, Rasmussen LS. Long-term consequences of postoperative cognitive dysfunction. Anesthesiology. 2009;110(3):548–55. 10.1097/ALN.0b013e318195b569. 19225398 10.1097/ALN.0b013e318195b569

[13] Greaves D, Psaltis PJ, Davis DHJ, et al. Risk factors for delirium and cognitive decline following coronary artery bypass grafting surgery: a systematic review and meta-analysis. J Am Heart Assoc. 2020;9(22):e017275. 10.1161/jaha.120.017275. 33164631 10.1161/JAHA.120.017275 PMC7763731

[14] Feinkohl I, Janke J, Slooter AJC, Winterer G, Spies C, Pischon T. Metabolic syndrome and the risk of postoperative delirium and postoperative cognitive dysfunction: a multi-centre cohort study. Br J Anaesth. 2023;131(2):338–47. 10.1016/j.bja.2023.04.031. 37344340 10.1016/j.bja.2023.04.031

[15] Feinkohl I, Winterer G, Pischon T. Diabetes is associated with risk of postoperative cognitive dysfunction: a meta-analysis. Diabetes Metab Res Rev. 2017;33(5):e2884. 10.1002/dmrr.2884. 10.1002/dmrr.2884 28063267

[16] Daiber A, Steven S, Weber A, et al. Targeting vascular (endothelial) dysfunction. Br J Pharmacol. 2017;174(12):1591–619. 10.1111/bph.13517. 27187006 10.1111/bph.13517 PMC5446575

[17] Forman DE, Cohen RA, Hoth KF, et al. Vascular health and cognitive function in older adults with cardiovascular disease. Artery Res. 2008;2(1):35–43. 10.1016/j.artres.2008.01.001. 21179381 10.1016/j.artres.2008.01.001 PMC3004172

[18] Wu MD, Atkinson TM, Lindner JR. Platelets and von Willebrand factor in atherogenesis. Blood. 2017;129(11):1415–9. 10.1182/blood-2016-07-692673. 28174163 10.1182/blood-2016-07-692673 PMC5356449

[19] Glumac S, Kardum G, Sodic L, et al. Longitudinal assessment of preoperative dexamethasone administration on cognitive function after cardiac surgery: a 4-year follow-up of a randomized controlled trial. BMC Anesthesiol. 2021;21(1):129. 10.1186/s12871-021-01348-z. 33892653 10.1186/s12871-021-01348-z PMC8063389

[20] Glumac S, Kardum G, Sodic L, Supe-Domic D, Karanovic N. Effects of dexamethasone on early cognitive decline after cardiac surgery: a randomised controlled trial. Eur J Anaesthesiol. 2017;34(11):776–84. 10.1097/eja.0000000000000647. 28985195 10.1097/EJA.0000000000000647

[21] Ottens TH, Dieleman JM, Sauer AM, et al. Effects of dexamethasone on cognitive decline after cardiac surgery: a randomized clinical trial. Anesthesiology. 2014;121(3):492–500. 10.1097/aln.0000000000000336. 25225745 10.1097/ALN.0000000000000336

[22] Román GC. Vascular dementia prevention: a risk factor analysis. Cerebrovasc Dis. 2005;20(Suppl 2):91–100. 10.1159/000089361. 16327258 10.1159/000089361

[23] Blann AD. A reliable marker of vascular function: does it exist? Trends Cardiovasc Med. 2015;25(7):588–91. 10.1016/j.tcm.2015.03.005. 25861960 10.1016/j.tcm.2015.03.005

[24] Castro-Ferreira R, Cardoso R, Leite-Moreira A, Mansilha A. The role of endothelial dysfunction and inflammation in chronic venous disease. Ann Vasc Surg. 2018;46:380–93. 10.1016/j.avsg.2017.06.131. 28688874 10.1016/j.avsg.2017.06.131

[25] Constans J, Conri C. Circulating markers of endothelial function in cardiovascular disease. Clin Chim Acta. 2006;368(1–2):33–47. 10.1016/j.cca.2005.12.030. 16530177 10.1016/j.cca.2005.12.030

[26] Groeneveld ON, van den Berg E, Johansen OE, et al. Oxidative stress and endothelial dysfunction are associated with reduced cognition in type 2 diabetes. Diab Vasc Dis Res. 2019;16(6):577–81. 10.1177/1479164119848093. 31068001 10.1177/1479164119848093

[27] Miralbell J, López-Cancio E, López-Oloriz J, et al. Cognitive patterns in relation to biomarkers of cerebrovascular disease and vascular risk factors. Cerebrovasc Dis. 2013;36(2):98–105. 10.1159/000352059. 24029412 10.1159/000352059

[28] Tomasi CD, Vuolo F, Generoso J, et al. Biomarkers of delirium in a low-risk community-acquired pneumonia-induced sepsis. Mol Neurobiol. 2017;54(1):722–6. 10.1007/s12035-016-9708-6. 26768428 10.1007/s12035-016-9708-6

[29] Huang CW, Tsai MH, Chen NC, et al. Clinical significance of circulating vascular cell adhesion molecule-1 to white matter disintegrity in Alzheimer’s dementia. Thromb Haemost. 2015;114(6):1230–40. 10.1160/th14-11-0938. 26289958 10.1160/TH14-11-0938

[30] Zuliani G, Cavalieri M, Galvani M, et al. Markers of endothelial dysfunction in older subjects with late onset Alzheimer’s disease or vascular dementia. J Neurol Sci. 2008;272(1–2):164–70. 10.1016/j.jns.2008.05.020. 18597785 10.1016/j.jns.2008.05.020

[31] Asif M, Soiza RL, McEvoy M, Mangoni AA. Asymmetric dimethylarginine: a possible link between vascular disease and dementia. Curr Alzheimer Res. 2013;10(4):347–56. 10.2174/1567205011310040001. 23036019 10.2174/1567205011310040001

[32] Pola R, Flex A, Gaetani E, et al. Association between intercellular adhesion molecule-1 E/K gene polymorphism and probable vascular dementia in humans. Neurosci Lett. 2002;326(3):171–4. 12095649 10.1016/s0304-3940(02)00337-3

[33] Quinn TJ, Gallacher J, Deary IJ, Lowe GD, Fenton C, Stott DJ. Association between circulating hemostatic measures and dementia or cognitive impairment: systematic review and meta-analyzes. J Thromb Haemost. 2011;9(8):1475–82. 10.1111/j.1538-7836.2011.04403.x. 21676170 10.1111/j.1538-7836.2011.04403.x

[34] Royall DR, Al-Rubaye S, Bishnoi R, Palmer RF. Serum protein mediators of dementia and aging proper. Aging (Albany NY). 2016;8(12):3241–54. 10.18632/aging.101091. 27922822 10.18632/aging.101091 PMC5270666

[35] Wolters FJ, Boender J, de Vries PS, et al. Von Willebrand factor and ADAMTS13 activity in relation to risk of dementia: a population-based study. Sci Rep. 2018;8(1):5474. 10.1038/s41598-018-23865-7. 29615758 10.1038/s41598-018-23865-7 PMC5882924

[36] Vasunilashorn SM, Ngo L, Inouye SK, et al. Cytokines and postoperative delirium in older patients undergoing major elective surgery. J Gerontol A Biol Sci Med Sci. 2015;70(10):1289–95. 10.1093/gerona/glv083. 26215633 10.1093/gerona/glv083 PMC4817082

[37] Menzenbach J, Frede S, Petras J, et al. Perioperative vascular biomarker profiling in elective surgery patients developing postoperative delirium: a prospective cohort study. Biomedicines. 2021;9(5):553. 10.3390/biomedicines9050553. 34063403 10.3390/biomedicines9050553 PMC8155907

[38] Mietani K, Sumitani M, Ogata T, et al. Dysfunction of the blood-brain barrier in postoperative delirium patients, referring to the axonal damage biomarker phosphorylated neurofilament heavy subunit. PLoS One. 2019;14(10):e0222721. 10.1371/journal.pone.0222721. 31574089 10.1371/journal.pone.0222721 PMC6771997

[39] Kearney-Schwartz A, Rossignol P, Bracard S, et al. Vascular structure and function is correlated to cognitive performance and white matter hyperintensities in older hypertensive patients with subjective memory complaints. Stroke. 2009;40(4):1229–36. 10.1161/strokeaha.108.532853. 19246701 10.1161/STROKEAHA.108.532853

[40] Markus HS, Hunt B, Palmer K, Enzinger C, Schmidt H, Schmidt R. Markers of endothelial and hemostatic activation and progression of cerebral white matter hyperintensities: longitudinal results of the Austrian Stroke Prevention Study. Stroke. 2005;36(7):1410–4. 10.1161/01.STR.0000169924.60783.d4. 15905468 10.1161/01.STR.0000169924.60783.d4

[41] Wada M, Takahashi Y, Iseki C, Kawanami T, Daimon M, Kato T. Plasma fibrinogen, global cognitive function, and cerebral small vessel disease: results of a cross-sectional study in community-dwelling Japanese elderly. Intern Med. 2011;50(9):999–1007. 10.2169/internalmedicine.50.4752. 21532222 10.2169/internalmedicine.50.4752

[42] Han JH, Wong KS, Wang YY, Fu JH, Ding D, Hong Z. Plasma level of sICAM-1 is associated with the extent of white matter lesion among asymptomatic elderly subjects. Clin Neurol Neurosurg. 2009;111(10):847–51. 10.1016/j.clineuro.2009.08.018. 19825506 10.1016/j.clineuro.2009.08.018

[43] Erdelyi-Botor S, Komaromy H, Kamson DO, et al. Serum L-arginine and dimethylarginine levels in migraine patients with brain white matter lesions. Cephalalgia. 2017;37(6):571–80. 10.1177/0333102416651454. 27206959 10.1177/0333102416651454

[44] Kant IMJ, de Bresser J, van Montfort SJT, Slooter AJC, Hendrikse J. MRI markers of neurodegenerative and neurovascular changes in relation to postoperative delirium and postoperative cognitive decline. Am J Geriatr Psychiatry. 2017;25(10):1048–61. 10.1016/j.jagp.2017.06.016. 28760515 10.1016/j.jagp.2017.06.016

[45] Kumon Y, Watanabe H, Tagawa M, Inoue A, Ohnishi T, Kunieda T. Relationship between deep white matter hyperintensities on magnetic resonance imaging and postoperative cognitive function following clipping of unruptured intracranial aneurysm. Neurol Med Chir (Tokyo). 2021;61(2):152–61. 10.2176/nmc.oa.2020-0290. 33390419 10.2176/nmc.oa.2020-0290 PMC7905299

[46] Winterer G, Androsova G, Bender O, et al. Personalized risk prediction of postoperative cognitive impairment - rationale for the EU-funded BioCog project. Eur Psychiatry. 2018;50:34–9. 10.1016/j.eurpsy.2017.10.004. 29398565 10.1016/j.eurpsy.2017.10.004

[47] Lammers F, Borchers F, Feinkohl I, et al. Basal forebrain cholinergic system volume is associated with general cognitive ability in the elderly. Neuropsychologia. 2018;119:145–56. 10.1016/j.neuropsychologia.2018.08.005. 30096414 10.1016/j.neuropsychologia.2018.08.005 PMC6338214

[48] Hayashi M, Kato M, Igarashi K, Kashima H. Superior fluid intelligence in children with Asperger’s disorder. Brain Cogn. 2008;66(3):306–10. 10.1016/j.bandc.2007.09.008. 17980944 10.1016/j.bandc.2007.09.008

[49] Feinkohl I, Borchers F, Burkhardt S, et al. Stability of neuropsychological test performance in older adults serving as normative controls for a study on postoperative cognitive dysfunction. BMC Res Notes. 2020;13(1):55. 32019577 10.1186/s13104-020-4919-3 PMC7001199

[50] Rasmussen LS, Larsen K, Houx P, Skovgaard LT, Hanning CD, Moller JT. The assessment of postoperative cognitive function. Acta Anaesthesiol Scand. 2001;45(3):275–89. 10.1034/j.1399-6576.2001.045003275.x. 11207462 10.1034/j.1399-6576.2001.045003275.x

[51] Duan MF, Zhu Y, Dekker LH, et al. Effects of education and income on incident type 2 diabetes and cardiovascular diseases: a dutch prospective study. J Gen Intern Med. 2022. 10.1007/s11606-022-07548-8. 35419742 10.1007/s11606-022-07548-8 PMC9640500

[52] Corley J, Gow AJ, Starr JM, Deary IJ. Smoking, childhood IQ, and cognitive function in old age. J Psychosom Res. 2012;73:132–8. 22789417 10.1016/j.jpsychores.2012.03.006

[53] Feinkohl I. Post-operative cognitive impairment: a cognitive epidemiology perspective. J Intelligence. 2022;10(1):18. 10.3390/jintelligence10010018 PMC8949407 35324574

[54] Borchers F, Rumpel M, Laubrock J, et al. Cognitive reserve and the risk of postoperative neurocognitive disorders in older age. Front Aging Neurosci. 2023;15:1327388. 10.3389/fnagi.2023.1327388. 38374990 10.3389/fnagi.2023.1327388 PMC10875020

[55] Jin Z, Hu J, Ma D. Postoperative delirium: perioperative assessment, risk reduction, and management. Br J Anaesth. 2020;125(4):492–504. 10.1016/j.bja.2020.06.063. 32798069 10.1016/j.bja.2020.06.063

[56] Leung JM. Postoperative delirium: are there modifiable risk factors? Eur J Anaesthesiol. 2010;27(5):403–5. 10.1097/EJA.0b013e3283340a99. 20386385 10.1097/EJA.0b013e3283340a99

[57] Daiello LA, Racine AM, Yun Gou R, et al. Postoperative delirium and postoperative cognitive dysfunction: overlap and divergence. Anesthesiology. 2019;131(3):477–91. 10.1097/aln.0000000000002729. 31166241 10.1097/ALN.0000000000002729 PMC6692220

[58] Huang H, Li H, Zhang X, et al. Association of postoperative delirium with cognitive outcomes: a meta-analysis. J Clin Anesth. 2021;75:110496. 10.1016/j.jclinane.2021.110496. 34482263 10.1016/j.jclinane.2021.110496

[59] Janelidze S, Mattsson N, Stomrud E, et al. CSF biomarkers of neuroinflammation and cerebrovascular dysfunction in early Alzheimer disease. Neurology. 2018;91(9):e867–77. 10.1212/wnl.0000000000006082. 30054439 10.1212/WNL.0000000000006082 PMC6133624

[60] Pappa M, Theodosiadis N, Tsounis A, Sarafis P. Pathogenesis and treatment of post-operative cognitive dysfunction. Electron Physician. 2017;9(2):3768–75. 10.19082/3768. 28465805 10.19082/3768 PMC5410904

[61] Cook-Mills JM, Marchese ME, Abdala-Valencia H. Vascular cell adhesion molecule-1 expression and signaling during disease: regulation by reactive oxygen species and antioxidants. Antioxid Redox Signal. 2011;15(6):1607–38. 10.1089/ars.2010.3522. 21050132 10.1089/ars.2010.3522 PMC3151426

