# Supplementary material for: The association of pre-operative biomarkers of endothelial dysfunction with the risk of post-operative neurocognitive disorders: results from the BioCog study
Source: BMC Anesthesiol. 2024 Oct 8;24:358. doi: 10.1186/s12871-024-02722-3 (PMC11459984; doi:10.1186/s12871-024-02722-3)
Supplement: Supplementary file 1 — Supplementary Material 1. [file 12871_2024_2722_MOESM1_ESM.docx]

**Supplementary figure 1.** Study Flow chart and selection of study population

**
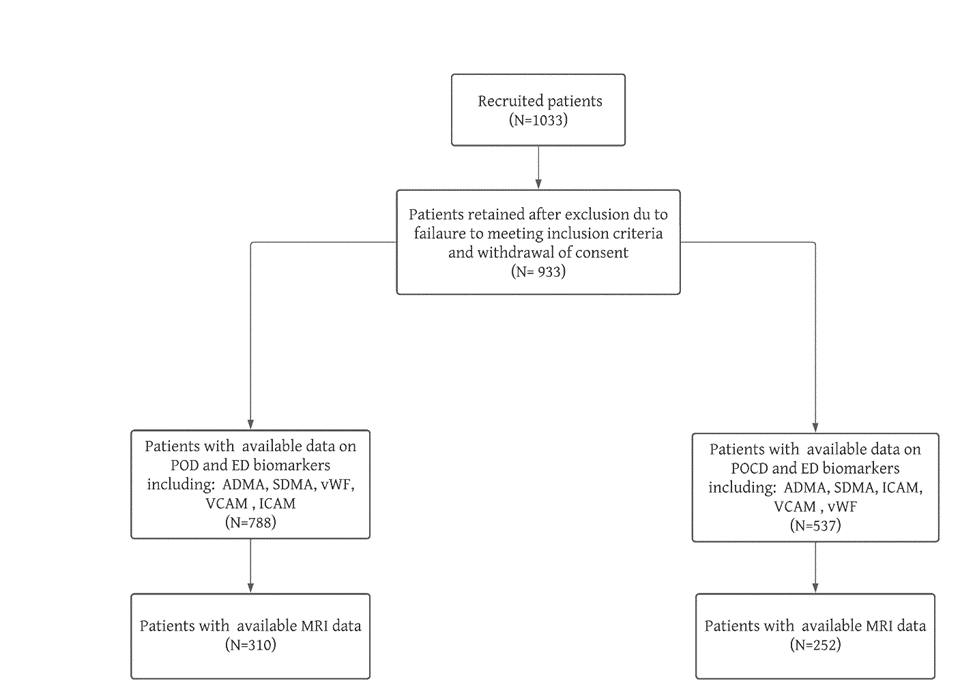
**

**Abbreviations:** Abbreviations: ADMA, asymmetric dimethylarginine; ICAM-1-1, intercellular adhesion molecule-1; MRI, magnetic resonance imaging; POD, post-operative delirium; POCD, post-operative cognitive dysfunction; SDMA, symmetric dimethylarginine; VCAM-1, vascular cell adhesion molecule-1; vWF, von Willebrand factor.

**Supplementary table 1A**. Participants sociodemographic and clinical characteristics at pre-operative assessment (before surgery) according to available data on POD development and ED biomarkers.

| Characteristics | Entire study population  (n = 933) | Study population with available data on POD and ED biomarkers  (n =788) | Study population without available data on POD and ED biomarkers  (n =145) |
| --- | --- | --- | --- |
| Age at inclusion. years (median, IQR)  Missing | 72.0 (68.0-76.0)  0 | 72.0 (68.0-76.0)  0 | 72.0 (68.0-76.0)  0 |
| Sex*(n, %)  Women  Missing | 395 (42.3)  0 | 336 (42.5)  0 | 59 (41.6)  0 |
| Surgery type (n, %)  Intracranial  Intrathoracic  Peripheral  Missing | 10 (1.1)  397 (43.5)  505 (53.4)  21 (2.2) | 10 (1.3)  340 (44.0)  422 (54.7)  16 (2.1) | -  57 (41,1)  82 (58.1)  6(4.1) |
| BMI, kg/m^2^ (median, IQR)  Missing (n, %) | 26.6 (24.0-29.4)  0 | 26.7 (24.1-29.4)  **0** | 26.3 (24.0-29.2)  **0** |
| Hypertension (n, %)  Missing | 589(63.1)  14 (1.5) | 498(63.0)  13 (1.60) | 91(64.1)  1 (0.7) |
| Diabetes (n, %)  Type 1  Missing  Type 2  Missing | 123(13.2)  14 (1.5)  182 (19.5)  23 (2.5) | 111 (14.0)  12 (1.5)  149 (18.8)  21 (2.6) | 12 (8.6)  2 (1.4)  33 (23.2)  2 (1.4) |
| History of stroke (n, %)  Missing (n, %) | 54 (5.8)  19 (2.0) | 44 (5.6)  17 (2.1) | 10 (7.0)  2 (1.4) |
| History of transient ischemic attack (n, %)  Missing (n, %) | 33 (3.5)  25 (2.7) | 29 (3.7)  22 (2.8) | 4 (2.8)  3 (2.1) |

* Significant difference between study population with and without available biomarker data (p<0.01). **Abbreviations**: IQR, Interquartile range; POD, post-operative delirium; ED; endothelial dysfunction.

**Supplementary table 1B**. Participants sociodemographic and clinical characteristics at pre-operative assessment (before surgery) according to available data on POCD development and ED biomarkers

| Characteristics ^1^ | Entire study population  (n = 933) | Study population with POCD and ED biomarkers  (n =537) | Study population without available data on POCD and biomarkers (n =396) |
| --- | --- | --- | --- |
| Age at inclusion.  years (median, IQR)  Missing (n, %) | 72.0 (68.0-76.0)  0 (0) | 72.0 (68.0-75.0)  0 (0) | 72.0 (69.0-76.0)  0 (0) |
| Sex (n, %) *  Women  Missing (n, %) | 395 (42.3)  0 (0) | 210 (38.75)  0 (0) | 142 (48.6)  0 (0) |
| Surgery type *  Intracranial  Intrathoracic  Peripheral  Missing (n, %) | 10 (1.1)  397 (43.5)  505 (53.4)  21 (2.2) | 5 (0.95)  222 (42.1)  301 (57.0)  7 (1.3) | 5 (1.7)  184 (46.5)  203 (51.2)  4 (1.4) |
| Hypertension (n, %)  Missing | 589(63.1)  14 (1.5) | 326 (61.4)  11 (1.7) | 198 (67.8)  3 (1.0) |
| History of transient ischemic attack  (n, %)  Missing (n, %) | 33 (3.5)  25 (2.7) | 25 (3.9)  20 (3.1) | 8 (2.7)  5 (1.7) |
| BMI, kg/m^2^ (median, IQR)  Missing (n, %) | 26.57 (24.0-29.3)  0 (0) | 26.57 (24.1-29.3)  0 (0) | 26.70 (23.7-29.8)  0 (0) |
| History of stroke (n, %)  Missing (n, %) | 54 (5.9)  19 (2.0) | 35 (5.5)  14 (2.6) | 19 (6.5)  4 (1.4) |
| Diabetes (n, %)  Type 2  Missing  Type 1  Missing | 182 (19.5)  14 (1.5)  123 (13.2)  14 (1.5) | 101 (19.1)  14 (2.3)  69 (12.9)  9 (1.7) | 56 (19.8)  8 (2.4)  47 (16.1)  4 (1.4) |

*Significant difference between study population with and without available biomarker data (p<0.01). **Abbreviations**: IQR, inter quartile range; POCD, post-operative cognitive dysfunction.

**Supplementary Table 2 A.** The correlations between participants sociodemographic and clinical characteristics and pre-operative (before surgery) ED biomarkers.

| Characteristics | ADMA (μmol/l) | SDMA (μmol/l) | ICAM-1 (ng/ml) | VCAM-1 (ng/ml) | vWF  (mU/ml) |
| --- | --- | --- | --- | --- | --- |
| Age (years)  Spearman correlation coefficient  p-value | 0.02  0.51 | 0.17  <0.01 | -0.02  0.47 | 0.11  <0.01 | 0.10  <0.01 |
| BMI (kg/m^2^)  Spearman correlation coefficient  p-value | -0.06  0.10 | -0.03  0.40 | 0.06  0.1 | 0.11  0.02 | 0.10  0.002 |
| MMSE Score  Spearman correlation coefficient  p-value | -0.06  0.10 | -0.12  0.02 | -0.06  0.06 | -0.12  <0.01 | -0.06  0.06 |
| g-Factor  Spearman correlation coefficient  p-value | 0.05  0.17 | 0.12  0.001 | 0.13  <0.01 | -0.14  <0.01 | -0.13  <0.01 |
| HbA1c (mmol/mol)  Spearman correlation coefficient  p-value | -0.04  0.25 | -0.04  0.25 | 0.08  0.03 | 0.03  0.50 | 0.13  0.01 |
| HDL (mmol/l)  Spearman correlation coefficient  p-value | 0.05  0.16 | -0.03  0.36 | -0.13  <0.01 | -0.14  <0.01 | -0.06  0.08 |
| IL-6 (pg/ml)  Spearman correlation coefficient  p-value | 0.04  0.20 | 0.05  0.18 | 0.13  <0.01 | 0.14  <0.01 | 0.16  <0.01 |
| ADMA (μmol/l)  Spearman correlation coefficient  p-value | 1.00  - | 0.32  <0.01 | 0.20  <0.01 | 0.25  <0.01 | 0.13  <0.01 |
| SDMA (μmol/l)  Spearman correlation coefficient  p-value | 0.32  <0.01 | 1  - | 0.14  <0.01 | 0.28  <0.01 | 0.24  <0.01 |
| ICAM-1 (ng/ml)  Spearman correlation coefficient  p-value | 0.20  <0.01 | 0.14  <0.01 | 1.00  - | 0.40  <0.01 | 0.36  <0.01 |
| VCAM-1 (ng/ml)  Spearman correlation coefficient  p-value | 0.25  <0.01 | 0.28  <0.01 | 0.40  <0.01 | 1.00  - | 0.32  <0.01 |
| vWF (mU/ml)  Spearman correlation coefficient  p-value | 0.13  <0.01 | 0.24  <0.01 | 0.36  <0.01 | 0.32  <0.01 | 1.00  - |

**Abbreviations**: ADMA, asymmetric dimethylarginine; BMI, body mass index; ED; endothelial dysfunction, ICAM-1-1, intercellular adhesion molecule-1; ICU, intensive care unit; IL-6, interleukin 6; IQR, Interquartile range; MMSE, mini-mental state examination; POD, post-operative delirium; SDMA, symmetric dimethylarginine; VCAM-1, vascular cell adhesion molecule-1; vWF, von Willebrand factor

**Supplementary table 2 B.** Concentrations of pre-operative (before surgery) ED biomarkers according to participants clinical characteristics.

|  | ADMA (μmol/l) | SDMA (μmol/l) | ICAM-1 (ng/ml) | VCAM-1 (ng/ml) | vWF  (mU/ml) |
| --- | --- | --- | --- | --- | --- |
| Sex  Female  Male  p-value | 0.80  0.77  0.13 | 0.77  0.81  0.13 | 667.70  667.82  0.89 | 875.92  881.88  0.71 | 1099.41  1012.88  0.30 |
| Hypertension  No  Yes  p-value | 0.79  0.78  0.27 | 0.76  0.81  0.05 | 656.77  674.63  0.22 | 848.68  899.63  0.30 | 993.67  1086.38  0.13 |
| Type 1 diabetes  No  Yes  p-value | 0.70  0.78  0.72 | 0.80  0.76  0.19 | 665.12  684.67  0.33 | 873.82  930.32  **0.02** | 1041.31  1095.84  0.85 |
| Type 2 diabetes  No  Yes  p-value | 0.78  0.80  0.24 | 0.78  0.83  0.33 | 664.14  683.77  0.21 | 870.83  923.08  **0.03** | 1023.44  1172.91  **0.01** |

^1^ P-value is driven by the Mann Whitney U test. **Abbreviations**: ADMA, asymmetric dimethylarginine; SDMA, symmetric dimethylarginine; VCAM-1, vascular cell adhesion molecule-1; vWF, von Willebrand factor.

**Supplementary Table 3 A.** The association betweenpre-operative (before surgery) cerebrovascular damage defined by MRI with pre-operative ED biomarkers in study participants.

| **Gray matter cerebral blood flow (arterial spin labeling) (ml/100g/min)** | **β coefficient^*^ (95% CI)** | **p-valve** |
| --- | --- | --- |
| **ADMA** | | |
| Model 1: unadjusted  Model 2: adjusted for age, sex, and surgery type | -0.001 (-0.16 to 0.17)  -0.01 (-0.19 to 0.14) | 0.97  0.65 |
| **SDMA** |  |  |
| Model 1: unadjusted  Model 2: adjusted for age, sex, and surgery type | -0.001( -0.14 to 0.13)  -0.01 (-0.14 to 0.12) | 0.78  0.54 |
| **VCAM-1** |  |  |
| Model 1: unadjusted  Model 2: adjusted for age, sex, and surgery type | 0.04 (-0.08 to 0.17)  0.03 (-0.09 to 0.17) | 0.50  0.52 |
| **ICAM-1** |  |  |
| Model 1: unadjusted  Model 2: adjusted for age, sex, and surgery type | 0.01 (-0.62 to 0.78)  0.005 (-0.19 to 0.21) | 0.83  0.91 |
| **VWF** |  |  |
| Model 1: unadjusted  Model 2: adjusted for age, sex, and surgery type | -0.07 (-0.12 to 0.02)  -0.10 (-0.12 to 0.02) | 0.19  0.15 |
| **The volume of WMH (mL)** | | |
| **ADMA** | | |
| Model 1: unadjusted  Model 2: adjusted for age, sex, and surgery type | 0.11 (0.02 to 1.18)  0.11 (0.05 to 1.17) | 0.04  0.03 |
| **SDMA** |  |  |
| Model 1: unadjusted  Model 2: adjusted for age, sex, and surgery type | 0.16 (0.24 to 1.19)  0.13 (0.12 to 1.05) | 0.003  0.01 |
| **VCAM-1** |  |  |
| Model 1: unadjusted  Model 2: adjusted for age, sex and surgery type | 0.08 (-0.08 to 0.81)  0.07 (-0.11 to 0.75) | 0.11  0.15 |
| **ICAM-1** |  |  |
| Model 1: unadjusted  Model 2: adjusted for age, sex and surgery type | 0.01 (-0.62 to 0.76)  0.01 (-0.60 to 0.75) | 0.83  0.82 |
| **VWF** |  |  |
| Model 1: unadjusted  Model 2: adjusted for age, sex and surgery type | 0.05 ( -0.13 to 0.37)  0.03 ( -0.15 to 0.33) | 0.36  0.47 |

*The β-coefficient is driven by Linear regression, β-coefficient refers to the increase in the mean of Gray matter cerebral blood flow and the volume of WMH per one standard deviation increase in levels of ED biomarkers.**Abbreviations:** ADMA, asymmetric dimethylarginine; CI, confidence interval; ICAM-1-1, intercellular adhesion molecule; OR, odds ratio; MRI, magnetic resonance imaging; SDMA, symmetric dimethylarginine; WMH, white matter hyperintensity; VCAM-1, vascular cell adhesion molecule-1; vWF, von Willebrand factor.

**Supplementary table 3 B**. The association between pre-operative cerebral infarctions defined by MRI and pre-operative ED biomarkers in study participants.

| **Cerebral infarctions** | **OR* (95% CI)** | **p-valve** |
| --- | --- | --- |
| **ADMA** | | |
| Model 1: unadjusted  Model 2: adjusted for age, sex, and surgery type | 0.21 (0.01 to 4.13)  0.19 (0.01 to 3.68) | 0.30  0.27 |
| **SDMA** |  |  |
| Model 1: unadjusted  Model 2: adjusted for age, sex, and surgery type | 1.45 (0.41 to 5.08)  1.46 (0.40 to 5.30) | 0.51  0.55 |
| **VCAM-1** |  |  |
| Model 1: unadjusted  Model 2: adjusted for age, sex, and surgery type | 0.99 (0.99 to 1.00)  0.99 (0.99 to 1.00) | 0.20  0.19 |
| **ICAM-1** |  |  |
| Model 1: unadjusted  Model 2: adjusted for age, sex, and surgery type | 0.99 (0.99 to 1.00)  0.99 (0.99 to 1.00) | 0.15  0.16 |
| **VWF** |  |  |
| Model 1: unadjusted  Model 2: adjusted for age, sex, and surgery type | 1.00 (0.99 to 1.00)  0.99 (0.99 to 1.00) | 0.50  0.52 |

*The odd ratio was driven by logistic regression. **Abbreviations:** Abbreviations: ADMA, asymmetric dimethylarginine; CI, confidence interval; ED, endothelial dysfunction; ICAM-1-1, intercellular adhesion molecule; OR, odds ratio; MRI, magnetic resonance imaging; SDMA, symmetric dimethylarginine; VCAM-1, vascular cell adhesion molecule-1; vWF, von Willebrand factor.
